# Adjuvant celecoxib and lanreotide following transarterial chemoembolisation for unresectable hepatocellular carcinoma: a randomized pilot study

**DOI:** 10.18632/oncotarget.15684

**Published:** 2017-02-24

**Authors:** Huan Tong, Bo Wei, Shuang Chen, Yong-Mei Xie, Ming-Guang Zhang, Lin-Hao Zhang, Zhi-Yin Huang, Cheng-Wei Tang

**Affiliations:** ^1^ Department of Gastroenterology, West China Hospital, Sichuan University, Chengdu, China; ^2^ Department of Pediatrics, West China Second University Hospital, Sichuan University, Chengdu, China

**Keywords:** hepatocellular carcinoma, transarterial chemoembolisation, celecoxib, lanreotide, survival

## Abstract

Recurrence of hepatocellular carcinoma (HCC) after transarterial chemoembolisation (TACE) is common due to neoangiogenesis. Cyclooxygenase-2 inhibitors and somatostatin analogues were reported to inhibit tumour angiogenesis. The pilot randomized controlled trial was aimed to prospectively evaluate the protocol of TACE combined with celecoxib and lanreotide (TACE+C+L) in patients with unresectable and advanced HCC. A total of 71 patients with HCC were enrolled and randomly assigned to either TACE (n=35) or TACE+C+L (n=36) group. Overall survival, disease control rate (DCR), and adverse events were assessed during a 3-year follow-up period. The median overall survival of the TACE+C+L group (15.0 months) was doubled compared to that of TACE group (7.5 months), *p* = 0.012. DCR of the TACE+C+L group was significantly higher than that of the TACE group either at 6 months (72.2% *vs* 42.9%, *p* = 0.012) or at 12 months (61.1% *vs* 28.6%, *p* = 0.006). The median overall survivals (13 months *vs* 4.5 months, *p* = 0.013) and DCR at 12 months (50% *vs* 13.6%, *p* = 0.008) of patients with advanced HCC in TACE+C+L groups were significantly higher than those in TACE group. No significant difference of adverse events was observed between the two groups. The occurrence of post-embolisation syndrome in TACE+C+L group was significantly lower than that in TACE group (16.7% *vs* 60.0%, *p* = 0.001). In conclusion, the regimen of TACE+C+L prolonged overall survival, enhanced tumour response, reduced post-embolisation syndrome and was well-tolerable in the patients with unresectable HCC. It may be more beneficial for advanced HCC.

## INTRODUCTION

As most of patients with hepatocellular carcinoma (HCC) are not suitable candidates for curative treatment, palliative treatment remains as the only available option. Transarterial chemoembolisation (TACE) is considered to be the first line therapy in patients with unresectable HCC of Barcelona Clinic Liver Cancer (BCLC) classified intermediate-stage (BCLC stage B) [1]. Despite the survival benefit of TACE in patients with intermediate HCC, TACE is a palliative treatment that does not result in complete tumor necrosis. Recurrence of HCC after TACE is common due to neoangiogenesis [2]. The neoangiogenesis of HCC after TACE has been linked to the increased expression of hypoxia-inducible factors or vascular endothelial growth factor (VEGF) in the residual surviving cancerous tissue [3, 4]. Theoretically, combination of TACE and antiangiogenic agents would be more feasible. However, combination strategies of TACE and systemic therapies (sorafenib or bevacizumab) have not resulted in clinical benefit [5, 6]. A meta-analysis that included the combination of different loco-regional therapies, such as TACE and radiofrequency ablation, produced some inconclusive results on TACE [7].

Cyclooxygenase (COX) is a key rate-limiting enzyme for the synthesis of prostaglandin. COX-2, an isoenzyme of COX, plays an essential role in the development of HCC. Over-expression of COX-2 has been considered as related to hepato-carcinogenesis and angiogenesis [8, 9]. Therefore, treatment with COX-2 inhibitors, such as celecoxib, might be promising in HCC. Previous studies have suggested that COX-2 inhibitors could inhibit the growth of HCC either in animal models or in patients [10, 11].

In addition, somatostatin is a widely distributed neuropeptide with multiple physiological functions, including immune regulation and tumour suppression. Overexpression of somatostatin receptors (SSTRs) has also been identified in HCC [12, 13]. Our previous experiment showed that somatostatin analogue (SSTA), such as octreotide and pasireotide, caused substantial necrosis of HCC xenografts *in vivo* [14]. Recently, we observed that pasireotide, combined with celecoxib, prolonged the survival in nude mice [15]. Moreover, combination of the above two types of non-cytotoxic agents displayed a synergistic inhibitory role on the growth or metastasis of the liver neoplasm in rabbits of post-TACE [16, 17]. However, the potential effects of the multi-modality protocol on the survival of patients who underwent TACE have not been assessed. As octreotide has a short half-life, lanreotide, the second SSTA available in the market, binds to the same receptors as octreotide with a higher affinity to SSTR-2, -5, and bears more advantages in the long-term therapy of HCC compared with octreotide. This prospective randomized controlled trial (RCT) was aimed to evaluate the overall survival, tumour response, disease control rate (DCR) and adverse events of TACE plus celecoxib and lanreotide in the patients with unresectable HCC.

## PATIENTS AND METHODS

### Patients

This RCT was conducted from October 2008 to June 2013 in West China Hospital, according to the protocol which conformed to the ethical guidelines of the Declaration of Helsinki 1975 and was approved by Chinese Ethics Committee of Registering Clinical Trials. The recruitment of participants ended in June 2010. The trial was registered in the Chinese Clinical Trial Registry (http://www.chictr.org/cn/; registration number: ChiCTR-TRC-08000191). Written informed consent was obtained from each patient.

Male or female patients between the ages of 18-75 years with a confirmed diagnosis of unresectable HCC were the eligible patients for this study. Patients with BCLC stage B-C [18] were recruited. Exclusion criteria were listed as below: decompensated liver disease (Child-Pugh grade B8-C15); active gastrointestinal bleeding; hepatic encephalopathy; extrahepatic metastasis; severe portosystemic shunt or hepatofugal blood flow; impaired clotting tests (platelet count < 50×10^9^/L or prothrombin activity < 50%); renal dysfunction; other severe systemic diseases; pregnant or breast feeding women and hyperallergy to drugs used in this study or to sulfanilamide.

### Grouping and intervention

Eligible patients were consecutively included and assigned to either TACE group or TACE+celecoxib+lanreotide (TACE+C+L) group, by using computer-generated randomization numbers. All clinical data were evaluated through history taking and clinical laboratory tests. Prior to the TACE procedure, liver function of each patient was evaluated according to the Child−Pugh grade. The general status of patients was evaluated using Karnofsky performance score (KPS). The patients were also stratified according to the BCLC stage for further analysis.

The schedule of TACE session was arranged as: baseline, 3 consecutive courses with interval of 2 months, then once per 3 months until the end of follow-up. Routine TACE procedure mainly included catheterization of flexible microcatheter (Terumo, Tokyo, Japan), injection of emulsion mixture with 20mg doxorubicin (Main Luck, Shenzhen, China) in 10ml iodized oil (Guerbet, Roissy CdG cedex, France) and embolisation with granules of absorbable gelatin sponge (Xiang’en, Nanchang, China).

Oral celecoxib (Celebrex **^®^**, Pfizer, New York, USA) 200 mg was given twice a day for 3 months, and afterwards 200 mg once daily until the end of follow-up. Lanreotide (Somatuline LA **^®^**, IPSEN, Paris, France) was administered as 40 mg intramuscular injections once monthly for 3 circles, then once per 2 months for 3 circles, and thereafter once per 3 months until the end of follow-up. Oral celecoxib 200 mg twice daily or lanreotide 40 mg intramuscular injections were initially administered a week prior to the first course of TACE.

TACE, administration of celecoxib and lanreotide would be discontinued if there were following settings: tumour response of progressive disease (PD) for > 6 months assessed by modified Response Evaluation Criteria in Solid Tumors (mRECIST) [19], liver function of Child−Pugh grade B8-C15, KPS<70, or any other item of the exclusion criteria. TACE would be withheld when viable tumour foci were not visible on imaging.

### Follow-up

The clinical course, DCR, tumour response and deaths were regularly documented per 3 months during the follow-up period of 3 years. Tumour response was assessed by contrast-enhanced ultrasound or spiral computed tomography, according to mRECIST [19], and two evaluators studied the imaging independently and were blinded to treatment assignment. Tumour response was defined into four grades: complete response (CR), partial response (PR), stable disease (SD), and PD. DCR was calculated as: (CR+PR+SD) / total patient number × 100%. Metastases were assessed by bone scintigraphy and cerebral MRI on the basis of the symptoms of patients. Endoscopy was performed when gastrointestinal bleeding was indicated. The adverse events were documented using Common Terminology Criteria for Adverse Events (CTCAE) version 3.0 at every visit [20]. The post-embolisation syndrome (PES) was set as a constellation of symptoms consisting of fevers, unremitting nausea, general malaise, loss of appetite, and variable abdominal pain following the TACE procedure.

### Efficacy outcomes

Overall survival was defined as the primary outcomes. The secondary outcomes included the following: tumour response, DCR, KPS and adverse events.

### Sample size calculation and statistical analysis

The sample size calculation was based on the assumption that there would be a median overall survival increase of 6 months from TACE treatment alone. A sample size of 28 cases in each group would provide a 90% power to estimate the overall survival improvement at 5% level of significance in this study. Taking into account a possibility of loss to follow-up of 20%, 35 cases were required in each group for this study.

Survival analysis was performed with the log-rank test. Cox proportional hazard regression was utilized to determine hazard ratios (HRs) of factors on survival. Univariate analysis and multivariate analysis were deployed to determine the influence of probable risk factors on survival. Similarly, the odds ratios (ORs) of factors on PES were calculated by logistic regression. Chi-square test was used for evaluating tumour responses. Baseline characteristics were tested using the chi-square or student's test, depending on the type of data. All the statistical work was conducted with SPSS 13.0 software (IBM, Chicago, USA).

## RESULTS

### Patient characteristics and compliance

**Figure 1:**
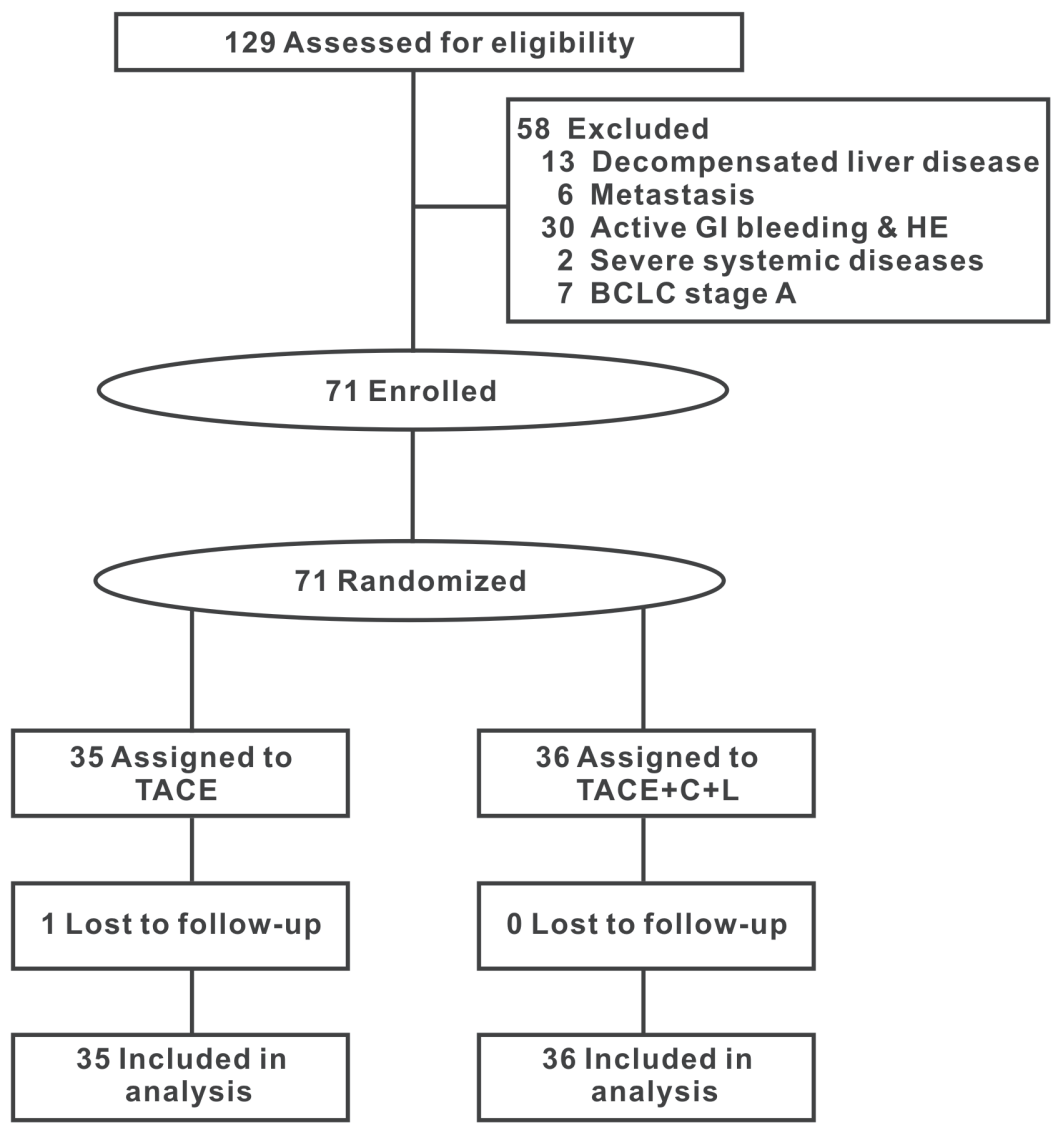
Flow of participants BCLC: Barcelona Clinic Liver Cancer; GI: gastrointestinal; HE: hepatic encephalopathy.

A total of 129 unresectable HCC patients were assessed for eligibility. Finally, 71 patients were enrolled sequentially, and one patient was lost during the follow-up (Figure 1). The baseline characteristics of two groups were in parallel (Table 1). Infection of hepatitis B virus was the major underlying liver disease of patients in both groups. The patients with BCLC stage C in the two groups were 2-fold of BCLC stage B. TACE group underwent 4.7± 4.0 sessions of TACE. In contrast, 7.5± 4.5 sessions of TACE were performed in TACE+C+L group, about 1.6-fold of TACE group.

**Figure 2:**
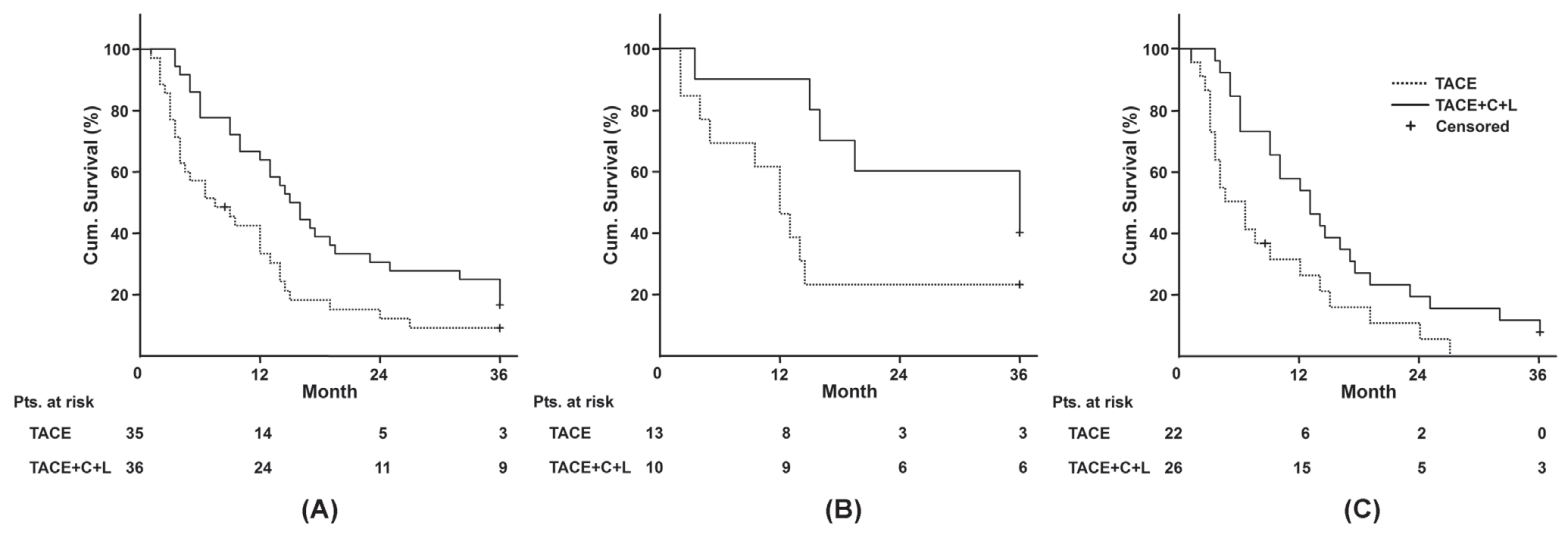
Evaluation of survival **A**. All patients; **B**. Patients with BCLC stage B; **C**. Patients with BCLC stage C. BCLC: Barcelona Clinic Liver Cancer; Cum.: Cumulative; Pts.: patients.

**Table 1:** Baseline characteristics

	TACE (*n* = 35)	TACE+C+L (*n* = 36)	*P* value
**Age (mean ± SD, years)**	55.4 ± 12.7	56.7 ± 12.4	0.664
**Gender (male/female, *n***)	29/6	31/5	0.705
**Underlying liver disease (*n***)			
Hepatitis B virus	24	23	0.828
Hepatitis C virus	0	1	
Alcohol	3	2	
Mixed	6	8	
Others	2	2	
With/without cirrhosis	30/5	27/9	0.257
**Child-Pugh grade A/B (*n***)	11/24	13/23	0.677
**AFP (< 400/> 400ng / ml, *n***)	16/19	18/18	0.718
**BCLC stage B/C (*n***)	13/22	10/26	0.399
**KPS (Mean ± SD)**	76.6 ± 7.3	78.1 ± 8.6	0.434
**Abdominal pain (*n***)	25	28	0.539
**Constitutional syndrome (*n***)^a^	31	30	0.735

^a^ Constitutional syndrome is characterized by the presence of asthenia, anorexia, malaise and weight loss.

AFP: alpha fetoprotein; BCLC: Barcelona Clinic Liver Cancer; KPS: Karnofsky performance score.

### Overall survival

The median overall survival of TACE+C+L group increased to 2-fold compared with TACE group (Table 2). The accumulated survival rate of patients in TACE+C+L group at 12 months (66.7%) was enhanced by 26.7%, compared with TACE group (40%), and the 24- and 36-month survival rates of TACE+C+L group (24-month, 30.6%; 36-month, 25%) were over 2-fold higher than those of TACE group (24-month, 14.3%; 36-month, 8.6%). The median overall survival (TACE+C+L *vs* TACE 13 months *vs* 4.5 months, *p* = 0.013) and DCR at 12 month (TACE+C+L *vs* TACE 50% *vs* 13.6%, *p* = 0.008) of patients with BCLC stage C in TACE+C+L group were significantly higher than those in TACE group. KPSs in TACE+C+L group both at 6 months and at 12 months were significantly higher than those in TACE group (Table 2). The constituent ratio of Child-Pugh grade B in TACE+C+L group at 12 months was significantly lower than that at baseline (at 12 months *vs* baseline 9/24 *vs* 23/36, *p* = 0.045). In contrast to it, TACE intervention alone did not improve the constituent ratio of Child-Pugh grade B at 12 month (at 12 months *vs* baseline 9/14 *vs* 24/35, *p* = 0.773). In addition, the rates of various causes of death were similar between two groups (Table 3).

**Table 2:** Overall survival, tumour response and life quality

	TACE	TACE+C+L	*P* value
**Overall survival (Median, 95% CI, months)**			
**All patients**	***n* = 35**	***n* = 36**	
	7.5 (2.4-12.6)	15.0 (12.1-17.9)	0.012
BCLC stage B	*n* = 13	*n* = 10	
	12.0 (7.9-16.1)	36.0 (11.0-61.1)	0.084
BCLC stage C	*n* = 22	*n* = 26	
	4.5 (1.7-7.3)	13.0 (8.0-18.0)	0.013
**KPS of all patients (Mean ± SD)**			
6-month	75.5 ± 7.6	81.3 ± 8.8	0.020
12-month	70.7 ± 6.2	81.3 ± 9.9	< 0.001
**Tumour response (CR/PR/SD/PD, n)**			
6-month			
All patients	0/6/9/5	0/17/9/5	0.220
BCLC stage B	0/4/4/1	0/7/2/0	0.289
BCLC stage C	0/2/5/4	0/10/7/5	0.305
12-month			
All patients	0/3/7/4	0/11/11/2	0.153
BCLC stage B	0/3/4/1	0/5/4/0	0.485
BCLC stage C	0/0/3/3	0/6/7/2	0.089
**DCR (%)**			
6-month			
All patients	42.9	72.2	0.012
BCLC stage B	61.5	90.0	0.179
BCLC stage C	31.8	65.4	0.020
12-month			
All patients	28.6	61.1	0.006
BCLC stage B	53.8	90.0	0.089
BCLC stage C	13.6	50.0	0.008

BCLC: Barcelona Clinic Liver Cancer; CR: complete response; DCR: disease control rate; KPS: Karnofsky performance score; PD: progressive disease; PR: partial response; SD: stable disease.

**Table 3:** Adverse events and cause of death

	TACE (*n* = 35)	TACE+C+L (*n* = 36)	*P* value
**Adverse events (*n***)			
Abdominal bloating (Grade 1)	12	5	0.044
Anorexia (Grade 1)	9	3	0.051
Diarrhea (Grade 1)	3	5	0.710
Fever (Grade 1)	7	3	0.189
Hemorrhage, GI (nonvariceal, Grade 1/2)	0/0	0/1	1
Liver dysfunction / failure (Grade 2/3)*	4/1	0/0	0.063
Nausea (Grade 1/2)	7/2	3/0	0.110
Pain (Grade 1/2)	2/3	1/0	0.157
Renal failure (Grade 3)^a^	1	1	1
Ulcer (Grade 1/2)	0/0	1/0	1
Vomiting (Grade 1/2)	5/1	1/1	0.218
**Cause of death (*n***)			
Liver failure^b^	15	11	0.282
VUGIB	7	7	0.953
HRS	4	2	0.429
Tumour rupture	4	3	0.710
Pneumonia	1	4	0.357

^a^ Adverse event grade 1 for liver dysfunction / failure and grade 1 and 2 for renal failure are not included in CTCAE version 3·0.

^b^ Deaths occur after the discontinuation of treatment for tumour response of progressive disease. The liver function does not deteriorate while treatment is discontinued.

HRS: hepatorenal syndrome; VUGIB: variceal upper gastrointestinal bleeding.

Univariate and multivariate analyses respectively showed that the HRs of probable risk factors on survival were as follows: BCLC stage 0.454 (95% CI 0.291 to 0.708, *p* = 0.001), 0.429 (95% CI 0.267 to 0.689, *p* < 0.001); KPS 0.941 (95% CI 0.913 to 0.971, *p*<0.001), 0.948 (95% CI 0.917 to 0.980, *p* = 0.001); celecoxib+lanreotide 0.534 (95% CI 0.321 to 0.888, *p* = 0.016), 0.485 (95% CI 0.283 to 0.831, *p* = 0.008). The HRs of age, gender and AFP level did not show significant impacts on the survival of patients underwent TACE, *p* > 0.05.

### Tumour response

No CR was observed in two groups. SD was the main tumour response (Table 2). DCR of the TACE+C+L group was significantly higher than that of the TACE group at either 6 months or at 12 months (Table 2). The significant difference was owing to the great enhancement of DCR in patients with BCLC stage C. DCRs of patients with BCLC stage C in TACE+C+L group increased to 2.1- and 3.7-fold of TACE group at 6 and 12 months, respectively.

### Adverse events

No significant difference of adverse events, including nonvariceal gastrointestinal hemorrhage, ulcer and liver dysfunction/failure, was observed between the two groups, except for abdominal bloating (Table 3). The several liver failure-related deaths in each group occurred after TACE, celecoxib and lanreotide were discontinued because of PD over 6 months. Also, no severe and fatal adverse events associated with celecoxib and lanreotide were observed in TACE+C+L group.

The occurrence of PES in TACE+C+L group was significantly lower than that in TACE group (TACE+C+L *vs* TACE, 16.7% *vs* 60.0%, *p* = 0.001). Univariate and multivariate logistic regressions respectively showed that the ORs for probable factors on PES were as follows: celecoxib+lanreotide 0.106 (95% CI 0.033 to 0.338, *p* < 0.001), 0.011 (95% CI 0.001 to 0.118, *p* < 0.001); age 2.212 (95% CI 1.233 to 3.968, *p* = 0.008), 3.250 (95% CI 1.529 to 6.910, *p* = 0.002); AFP 0.427 (95% CI 0.191 to 0.954, *p =* 0.038), 0.313 (95% CI 0.114 to 0.861, *p =* 0.024). The ORs of gender, BCLC stage, KPS and TACE sessions did not show significant impacts on PES of patients underwent TACE, *p* > 0.05.

## DISCUSSION

The global burden of HCC is increasing. RCTs to test new treatments in the adjuvant setting and to test combination therapies in intermediate and advanced HCC are main medical needs for HCC. However, none of the combination therapies with chemoembolisation has shown additive outcome advantages. This is the first clinical trial of a systemic utilization of celecoxib and lanreotide in combination with TACE (TACE+C+L). Compared with TACE group, TACE+C+L group significantly increased the median overall survival by 7.5 months (from 7.5 months to 15 months). Among the probable risk factors on survival, BCLC stage, KPS and celecoxib+lanreotide showed significant HRs. The 2-fold higher overall survival in TACE+C+L group indicated the adjuvant efficacy of celecoxib and lanreotide when the baselines of BCLC stage and KPS were in parallel.

The survival of HCC patients is rationally related to tumour progress and cirrhotic improvement. DCRs of TACE+C+L group were significantly higher than that of TACE group at both 6 months and 12 months, suggesting a better tumour response for TACE+C+L regimen. Such a synergistic inhibitory role in HCC treatment is supported by the basis of our previous experiment of rabbits with hepatic VX2 allografts. In the animal model, TACE plus octreotide and celecoxib synergistically prolonged the survival through antiangiogenesis, induction of allograft capsule formation, and inhibition of growth and metastasis of the tumour [16, 17]. Although protocol of either TACE+lanreotide/octreotide or TACE+celecoxib ever showed a little survival benefit in our previous animal experiment or preclinical test, their efficacies were inferior to that of TACE+lanreotide/octreotide+celecoxib. The potential mechanisms behind the regimen may due to the inactivation of p-ERK - HIF-1α/VEGF, endothelial nitric oxide synthase, MAPK - ERK, JNK - p38, and TGF-β1/Smads integrated signaling pathways, which are involved in the cross-talk of COX-2 and SST signal transduction. The synergistic outcome of SSTA and COX-2 inhibitor led to a higher efficiency in inhibition of p-ERK, HIF-1α, VEGF and angiogenesis than either one alone [15–17, 21–23].

As the tumour microenvironment has a crucial role in the natural history of HCC, there is a strong rationale for anti-inflammation and reduction of pro-tumorigenic stroma. About 80% of patients in this study had concomitant cirrhosis. Besides chronic inflammation plays a stimulatory role in the fibrosis, proinflammatory conditions promote HCC onset and progression via activation of Wnt and EGFR signaling pathways [24]. Celecoxib and octreotide may ameliorate liver fibrosis in the cirrhotic rats model via the inhibition of intrahepatic and extrahepatic inflammation, and angiogenesis [23, 25, 26]. Also, celecoxib could inhibit the epithelial-mesenchymal transition of hepatocytes by reduction of intrahepatic inflammation, preservation of normal basement matrix and inhibition of TGF-β1/Smad pathway [27]. Besides anti-fibrosis, the pharmacological effect of lanreotide on portal hypertension through reduction of blood volumes in the portal system allows sufficient perfusion in vital organs, reducing the risk of ascites and the hepatorenal syndrome [28]. Therefore, the combination of celecoxib and lanreotide may be helpful to target the microenvironment in HCC. The greatly enhanced KPS and decreased ratio of Child-Pugh grade B in TACE+C+L group suggested the improvement of liver cirrhosis.

The short half-life period (8 h) of octreotide becomes a drawback in the long term treatment of HCC. As a member of somatostatin analogue family, lanreotide in a slow release formulation (7-14 d) was originally indicated for acromegaly and gastroenteropancreatic neuroendocrine tumors [29]. The administered schedule in this setting is recommended as 40 mg intramuscularly every 2 weeks. Although there are few case reports on the application of lanreotide in HCC treatment, no recommendation of dosage or interval was available for lanreotide (Somatuline LA **^®^**) in HCC treatment [30, 31]. In this study, the down-stair interval of lanreotide injection was on the basis of following considerations: 1) Conventional injection interval of lanreotide was on the basis of antisecretory efficacy of its pharmacokinetic profile. This might be unfavorable interval of antitumoral effects on account of complicated mechanisms. 2) A rational administration interval of lanreotide during the prolonged HCC treatment should consider the maintenance of sensitivity of SSTRs and reduction of adverse events. Persistent administration of high dosage of SSTA may induce receptor internalization, which renders the target cell insensitive to SSTA [32, 33]. Although lanreotide was tolerable in most of clinical studies less than 1 year, diarrhea and gallstone formation were identified as the main adverse events. Impairment of glucose homeostasis was a regular phenomenon. Our preclinical tests with several administration interval of lanreotide were much helpful to set the regimen of lanreotide for the pilot RCT.

The BCLC staging system provides an easy-to-use algorithm that links tumour stages with treatment allocation policies based on evidence [1, 34]. BCLC stage B is indicated to TACE, the standard of care with a median survival of 26 months for intermediate HCC [35], while sorafenib represents the first line treatment with a median survival of 6.5 months in HCC patients with BCLC stage C, the advanced HCC [36]. However, a growing body of evidence supports the use of TACE for patients with early and advanced HCC [37, 38] because of the tumour heterogeneity with more important therapeutic implications. Even if portal invasion is demonstrated, it is not the absolute contraindication of TACE [39]. The applications of surgical resection plus TACE and surgical resection plus sorafenib were the alternative strategies for BCLC stages B and C [7]. Therefore, patients with either BCLC stage B or C were mixed into two groups of this study. More advanced HCC in both groups resulted in lower median overall survival (7.5 months in TACE group and 15 months in TACE+C+L group). With stratified evaluation, the median overall survival and DCR at 12 months of patients with BCLC stage C in TACE+C+L group were significantly higher than those in TACE group, suggesting that the protocol of TACE+C+L may be an alternative strategy for the patients with advanced HCC.

PES is a common complication caused by intrahepatic and extrahepatic inflammation due to tumor ischemia. By means of their anti-inflammatory characteristics, celecoxib+lanreotide significantly reduced the occurrence of PES with ORs of 0.011 (*p* < 0.001). It would be beneficial especially for aged patients due to ORs of age being 3.250 (*p* = 0.002). Although the symptoms of PES are usually self-limited, the severe complication may drive the cirrhotic stage into worse one and even liver failure [40]. The 43.3 % reduction of PES in TACE+C+L regimen would be positive for TACE compliance of HCC patients, which in turn endued more opportunities to undergo TACE sessions and improved DCR.

More mild abdominal bloating was complained in TACE group probably due to untreated PES. Other adverse events of the two groups may be partly or temporally related to TACE or celecoxib and lanreotide. The adverse events during long term administration of celecoxib and lanreotide are an issue of concern. Fortunately, the adverse events in TACE+C+L regimen were not more than those of TACE alone and no special adverse events were associated with celecoxib and lanreotide. All of patients in TACE+C+L group completed the follow-up with good compliance. Compared to sorafenib treatment, the dermatological reactions, fatigue, diarrhea, and arterial hypertension associated with sorafenib usually compel dose reduction or interruption of treatment [41].

This study has several limitations. Firstly, this is a study conducted in a single center. Secondly, this study is neither single- nor double-blinded which might introduce bias. A well-designed multiple centers RCTs are necessary to control the bias and afford better evidence.

In conclusion, the novel regimen of TACE with adjuvant celecoxib and lanreotide prolonged overall survival, enhanced tumour response, reduced PES and was well-tolerable in the patients with unresectable HCC. It may be more beneficial for the patients with advanced HCC.
